# Neurofeedback therapy to improve cognitive function in patients with chronic post-stroke attention deficits: a within-subjects comparison

**DOI:** 10.3389/fnhum.2023.1155584

**Published:** 2023-07-11

**Authors:** Sonja C. Kleih-Dahms, Loic Botrel

**Affiliations:** Department of Psychology I, Section Intervention Psychology, Institute of Psychology, University of Würzburg, Würzburg, Germany

**Keywords:** stroke, slow cortical potentials (SCP), attention deficit, neurofeedback training, self-efficacy, brain-computer interfaces (BCI)

## Abstract

**Introduction:**

We investigated a slow-cortical potential (SCP) neurofeedback therapy approach for rehabilitating chronic attention deficits after stroke. This study is the first attempt to train patients who survived stroke with SCP neurofeedback therapy.

**Methods:**

We included *N* = 5 participants in a within-subjects follow-up design. We assessed neuropsychological and psychological performance at baseline (4 weeks before study onset), before study onset, after neurofeedback training, and at 3 months follow-up. Participants underwent 20 sessions of SCP neurofeedback training.

**Results:**

Participants learned to regulate SCPs toward negativity, and we found indications for improved attention after the SCP neurofeedback therapy in some participants. Quality of life improved throughout the study according to engagement in activities of daily living. The self-reported motivation was related to mean SCP activation in two participants.

**Discussion:**

We would like to bring attention to the potential of SCP neurofeedback therapy as a new rehabilitation method for treating post-stroke cognitive deficits. Studies with larger samples are warranted to corroborate the results.

## 1. Introduction

Stroke is the second leading cause of death worldwide, responsible for approximately 11% of all deaths (WHO, 2020). In people who survive a stroke, not only motor disability is a common consequence but also cognitive deficits (Pollock et al., 2014). Post-stroke attention deficits appear frequently (Hochstenbach et al., 1998). At the same time, it remains uncertain whether cognitive rehabilitation approaches improve attention deficits in the long term (Loetscher et al., 2019). According to Niemann and Gauggel (2010), attention summarizes several abilities, such as alertness, sustained attention, divided attention, and executive attention. Some or all these aspects of attention may be affected in people who survived a stroke, affecting higher-order cognitive processes and preventing patients from successful participation in rehabilitation treatment (Van Gilder et al., 2020) with far-reaching consequences. Adherence to physical activity programs significantly correlated with functional recovery 18 months after stroke (Gunnes et al., 2019). A lack of recovery prevents successful and satisfying participation in societal activities (Kapoor et al., 2019) and negatively affects wellbeing and quality of life (Ones et al., 2005). Quality of life might also be negatively affected by the inability to perform instrumental activities, such as preparing a meal or driving a car (Kapoor et al., 2019). Functional independence and the ability to engage in activities of daily living were significantly negatively influenced by depression (Ezema et al., 2019). Cognitive deficits correlated significantly with symptoms of depression 6 months post-stroke (Williams and Demeyere, 2021), which stresses the importance of treating cognitive deficits to improve motor functioning and prevent emotional distress or even the development of clinical depression (Torrisi et al., 2018). Torrisi et al. (2018) pointed out that high perceived self-efficacy (Bandura, 1977) was more prominent in patients with better functional recovery and recommended interventions to strengthen self-efficacy. Training to improve perceived self-efficacy (Abolfathi et al., 2018) increased subjectively reported quality of life in hospitalized patients after stroke. Another study pointed out the relationship between self-efficacy and wellbeing, illness acceptance, functional status in basic activities of daily living, and locomotive abilities (Szczepańska-Gieracha and Mazurek, 2020). In their review on self-efficacy and self-management after a stroke, Jones and Riazi (2011) supported the assumption that self-efficacy plays a role in stroke rehabilitation. They reported self-efficacy to be related to quality of life, activities of daily living, and depression. Self-efficacy is strengthened most by mastery experiences (Bandura, 1977), which means the experience of control but also of being able to learn and master a task that might have been difficult to learn. Neurofeedback is a self-regulation task for which learning to control brain activation is necessary (Davelaar, 2018). After a consolidation phase, a sense of automatic mastery might occur, strengthening self-efficacy beliefs. In support of this assumption, a beneficial effect of neurofeedback on self-efficacy was found (Harris et al., 2021).

Compared to other rehabilitation approaches, slow cortical potential neurofeedback (SCP NF) aims at a change in brain activation and thereby causes a behavior change (Hammond, 2011). In NF approaches, brain activity is assessed via electrodes on the scalp. The activity is fed back to the user mainly by an object manipulated in correspondence to one specific brain activity (Strehl, 2013). As changes to the object are linked to this specific brain activity of interest, it is possible to learn to influence brain activation (Davelaar, 2018). If the NF training is successful, a behavioral change occurs in correspondence to the trained brain activity. SCPs are event-related potentials that last up to several seconds (Elbert, 1993), while negative activation shifts represent cortical activation (Pribram and McGuinness, 1975) and positive shifts reflect cortical inhibition (Elbert, 1993). Slow cortical potentials with a negative polarity were hypothesized to facilitate information processing in the brain areas where the processing will occur (Rockstroh et al., 1984). Rockstroh et al. (1984) stated that stimulus selection is one essential ability of attention to be regulated by the mediothalamic-frontocortical system, which also is one generator of SCPs (Rockstroh et al., 1984). More specifically, SCP regulation activates the precentral gyrus, the thalamus, the inferior frontal gyrus, and the supplementary motor area (Hinterberger et al., 2005). There is evidence that the thalamus does play a role in selective attention (Tokoro et al., 2015; Wimmer et al., 2015). Accordingly, SCP negativity training could contribute to an improvement in attention. Shifting SCP activity toward negativity improved reaction times in healthy subjects (Lutzenberger et al., 1979) and reduced symptoms in clinical populations. Children with Attention Deficit Hyperactivity Syndrome increased cortical excitability via SCP NF toward negativity and decreased attention deficits (e.g., Arns et al., 2009). Patients with epilepsy learned to increase SCP shifts toward positivity linked to cortical inhibition, and the number of seizures decreased (Kotchoubey et al., 1997).

In this study, we investigated patients with chronic stroke. While, to our knowledge, SCP NF was not applied to patients with chronic stroke yet, several researchers showed that regulation of other EEG frequencies was possible and improved cognitive functions such as memory and attention (Doppelmayr et al., 2007; Kober et al., 2015, 2017; Reichert et al., 2016; Mottaz et al., 2018).

We hypothesized that participants would learn to regulate their slow-cortical potentials toward negativity (H1), thereby increasing their attention performance (H2). We hypothesized neurofeedback training to improve emotional state, self-efficacy, and quality of life, and increase competence in the performance of activities of daily living in those patients who successfully improved attention (H3). As we found self-regulated learning of brain activation changes associated with motivation (Kleih-Dahms et al., 2021), we hypothesized better SCP regulation related to higher motivation (H4).

## 2. Methods

### 2.1. Design

We implemented a within-subjects follow-up design. Dependent variables were SCP negativity mean amplitudes and the results of neuropsychological and psychological tests. Primary outcome variables were increased SCP negativity and an improvement in attention. Secondary outcome variables were improvements in psychological aspects, as these were hypothesized to depend on improving attention. Participants were tested 4 weeks before SCP training (t0), in the week before the SCP training (t1), in the week after the SCP training (t2), and in a 3-month follow-up assessment (t3).

### 2.2. Participants

Inclusion criteria were a stroke event more than half a year ago (chronic state), subjectively reported attention deficits during daily activities, a reported history of neuropsychological deficits, and commitment toward participation in a time-intense neurofeedback training. We calculated the necessary sample size for this study using the G^*^Power software tool (version 3.1) by Faul et al. (2007). Based on a medium effect size (0.3), α = 0.05, β = 0.80, four assessments per participant, and a repeated measure within factors test, a sample size of *N* = 17 was calculated. Nevertheless, we aimed at a total sample size of *N* = 20. Unfortunately, due to the COVID-19 pandemic, we could not accomplish that goal. Therefore, we included *N* = 8 patients; *n* = 3 dropped out due to either beginning medication with neuroleptics during participation in the study (*n* = 1), the occurrence of another stroke event (*n* = 1), or diagnosis with epilepsy throughout participation in the study (*n* = 1). The remaining sample consisted of *n* = 5 participants, *n* = 3 participants were male, and the mean age was 68.40 years (*SD* = 5.43). We summarized details about the participants and diagnoses in Table 1. Every participant was reimbursed 100 € for travel expenses. The Medical Ethics Committee of the University of Würzburg approved the study.

**Table 1:** Participant number (PN), sex (f, female; m, male), age, lesion side, medical description of the stroke, and years since the stroke event (=ysse) were displayed for every participant.

**PN**	**Sex**	**Age**	**Lesion side**	**Medical description**	**Ysse**
01	F	60	Left	Middle cerebral artery infarction	1
03	M	69	Left	Left-sided thalamus infarction	3
06	M	70	Right	Carotis interna artery infarction	14
07	F	68	Left	Multiple left-sided lacunar infarctions with localization in the semioval center	7
08	M	76	Bilateral	Multiple right and left hemispheric lacunar infarctions	13

### 2.3. Procedure and stimuli

**Figure 1:**
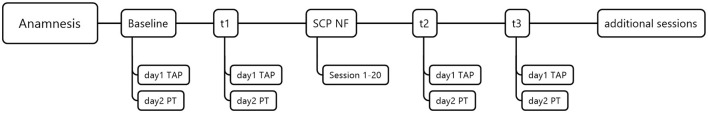
Procedure. TAP, test battery for attention performance; PT, psychological tests; SCP NF, Slow-cortical potential neurofeedback; and additional sessions with therapeutic content addressing various needs and problems.

Neuropsychological and psychological tests were assessed on two separate days, each lasting about 2 h. The 20 SCP neurofeedback therapy sessions lasted approximately 1 h each, including preparation time and instructions. With the anamnesis, baseline, t1, 20 neurofeedback therapy sessions, t2, t3, and follow-up assessment, each participant underwent 29 appointments (see Figure 1). Participant 07 underwent 14 out of 20 neurofeedback sessions due to a required surgery. In addition, three participants requested additional appointments to address psychological post-stroke challenges beyond SCP neurofeedback therapy (e.g., relationship with a spouse, low self-esteem, and changing roles in the family). Psychological treatment was based on the systemic therapy approach, took place after the follow-up assessments, and is not part of any study.

#### 2.3.1. Neuropsychological tests

We measured attention using the test battery for attention performance (TAP, Zimmermann and Fimm, 2012). We used the subtests alertness, divided attention, sustained attention, and go/NoGo tests.

##### 2.3.1.1. TAP alertness

The *TAP Alertness* test examines intrinsic and phasic alertness. To assess intrinsic alertness (trials one and four), participants press a reaction key as fast as possible if a cross appears on the screen. To assess phasic alertness, a warning tone precedes the cross stimulus (trials two and three). Each trial includes 20 target stimuli. Our dependent variable was reaction time.

##### 2.3.1.2. The TAP divided attention test

In the *TAP Divided Attention* (auditory + visual) test, the participant is instructed to attend to a visual and an auditory task simultaneously. In the visual task, a pattern of four crosses forming a square must be detected, and a key press is required. The crosses randomly change positions on a predefined matrix. One hundred stimuli are presented, 16 of which are critical. In the auditory task, participants must react with a key press in case two tones of the same frequency are subsequently presented. Two hundred trials, including 16 critical ones, are presented. As reaction times are less indicative of the ability for divided attention than omissions and errors (Zimmermann and Fimm, 2012), those were our dependent variables.

##### 2.3.1.3. The TAP sustained attention task

In this test, 450 figures that vary in shape, color, size, and filling are presented on a black computer screen. Fifty-four stimuli are critical. The test duration is 15 min. The participant needs to press the reaction key when the shape or color of a presented figure corresponds to the previously presented figure. Other figure characteristics are irrelevant and should be ignored. The relevant dependent variables in this subtest were reaction time, number of errors, and number of omissions.

##### 2.3.1.4. The TAP Go/NoGo task

We used the “1 out of 2” version of this test in which a cross (x = critical stimulus) and a standing cross (+) are presented. A key press is required after the presentation of the cross. A total of 40 stimuli are presented, of which 20 are critical. Dependent variables were reaction times, errors, and omissions.

#### 2.3.2. Psychological questionnaires

##### 2.3.2.1. Allgemeine Depressionsskala Langform

To measure possible depressive symptoms in participants, we used the German version of the *Center for Epidemiological Studies Depression Scale—the Allgemeine Depressionsskala Langform* (ADS-L, Hautzinger et al., 2012). The ADS-L is used mainly in non-clinical populations to assess the presence and duration of depression symptoms, motor inhibition, and harmful thought patterns. The ADS-L contains 20 items to be rated on a 4-point Likert scale ranging from 0 (“rarely or never”) to 3 (“most of the time or always”). Higher scores indicate more severe symptoms. The cutoff value is 23.

##### 2.3.2.2. The Aachen self-efficacy scale (ASS, German: Aachen Selbstwirksamkeitsfragebogen)

The ASS (Wälte and Kröger, 1995) measures self-efficacy in the three domains of work/performance, interaction, and body/health using 20 items to be rated on a 5-point Likert scale ranging from does not apply at all (=1) to applies completely (=5). Internal consistency is high with 0.91 re-test reliability ranges between 0.59 and 0.63. Higher values indicate better self-efficacy.

##### 2.3.2.3. The Aachener functioning item bank (AFIB, German: Aachener Funktionsitembank)

For assessing activities of daily living (ADL), we used the AFIB, an item pool instrument based on the Rasch model (Böcker, 2013). Daily functioning comprises three different areas: (1) applied cognition, (2) personal care, social activities (inclusion), and (3) physical and movement activities (mobility). In this study, we focused on the cognition scale including 18 items (e.g., “During the last days, I was able to focus on the essentials without help.”) to be answered on a 5-point Likert scale ranging from “0” (I was completely unable to do that) to “4” (without difficulty). The Rasch transformation values range from −5.802, which indicates the lowest possible ability to perform activities of daily living, to 5.802, which indicates the highest possible ability.

##### 2.3.2.4. The short form-36 health survey

The SF-36 (Morfeld et al., 2012) assesses subjective health using 35 items of eight subscales representing subjective health. These eight dimensions are physical functioning, role-physical, bodily pain, general health, vitality, social functioning, role-emotional, and mental health. The physical functioning sum score is based on 10 items, while the mental health sum score comprises five. These two sum scores were our dependent variables. Internal consistency of the SF-36 subscales is mostly 0.70 or above except for the subscales health and social functioning (0.57 and 0.69). Re-test reliability was, on average, 0.75 and therefore is satisfying. Raw values were transformed to range between 0 (worst possible quality of life) and 100 (best possible quality of life).

##### 2.3.2.5. Visual analog scales measuring motivation and attention

The visual analog scales (VAS) motivation and attention ranged from 0 (not motivated at all, not feeling attentive at all) to 10 (very motivated, feeling highly attentive) on a 10-cm horizontal line.

#### 2.3.3. SCP neurofeedback

The Thera Prax Mobile^®^ System (Neurocare group Munich, Germany) controlled SCP neurofeedback. Participants were seated 50 cm from a computer screen (15”, 1024 × 768). The neurofeedback therapist and experimenter sat at a separate table following the training on a separate trainer screen. The therapist delivered social feedback to support learning beyond the learning effects caused by computer-based feedback. The therapist motivated the participants during the training and between runs and informed them about artifacts in the EEG caused by muscle tension. Before the SCP neurofeedback started, possible eye movement artifacts were assessed in a structured protocol with three trials (40 s each) and various instructions (e.g., “look up and down,” “look left and right”). Eye movements were assessed using four electrodes, one above and one below the left eye and two next to the outer canthi of both eyes. During SCP training, the artifacts produced by eye movement were online corrected (Croft and Barry, 2000), while uncorrected data were saved. Additionally, trials with potential shifts above 200 μV were aborted, deleted, and repeated at the end of the training run.

**Figure 2:**
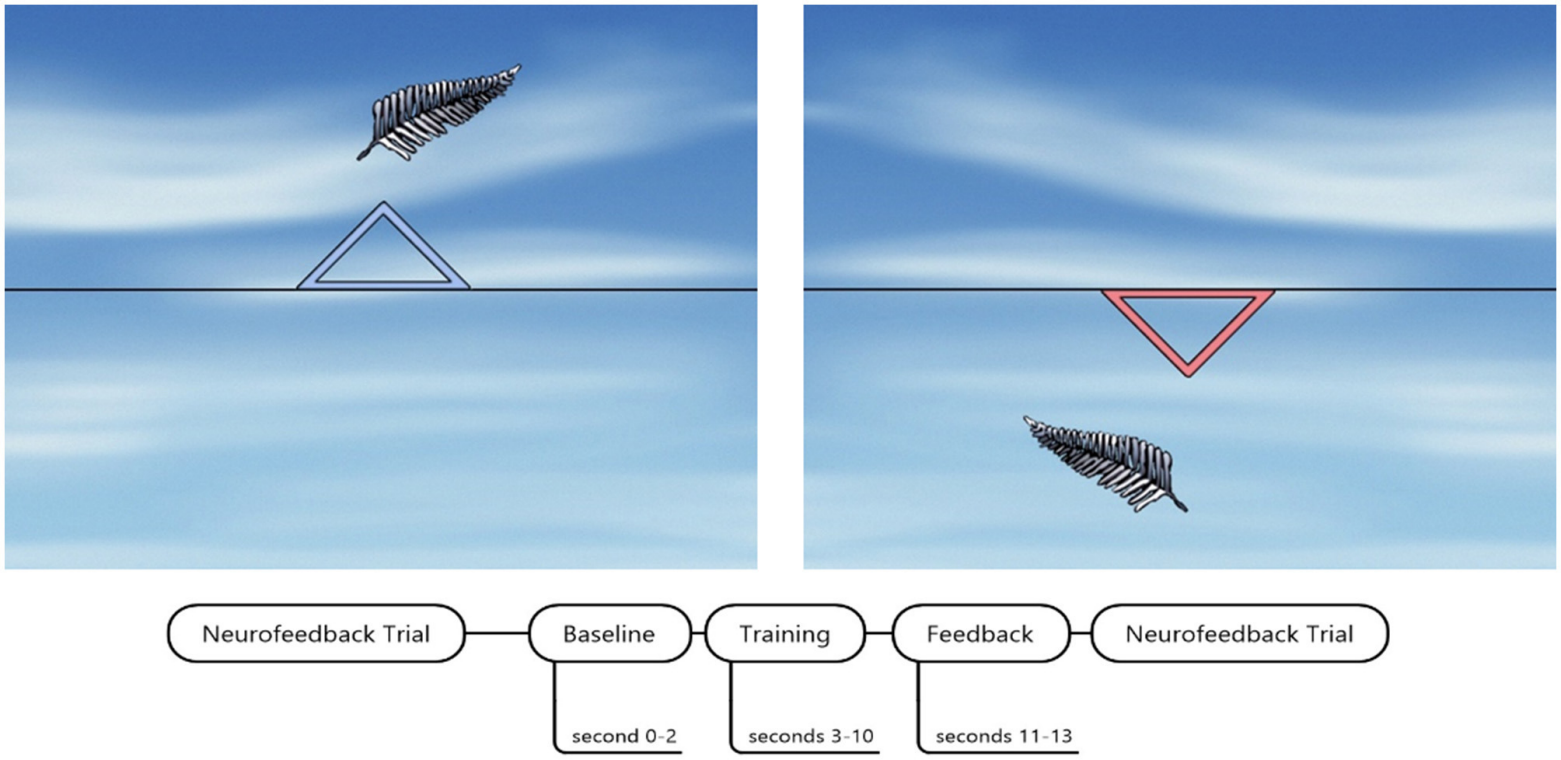
SCP neurofeedback screen and timing of one trial. The blue triangle indicated SCP regulation toward cortical negativity. The red triangle down indicated SCP regulation toward cortical positivity. SCP neurofeedback screen by courtesy of neurocare group Munich, Germany.

Every SCP neurofeedback session consisted of four runs, including 40 trials each (for trial structure see Figure 2). Each run lasted 8 min. On the participant screen, a black line was presented on a background imitating a blue sky with clouds (see Figure 2). The black line marked baseline EEG activation. A colored triangle appeared at the beginning of each trial, indicating either the task to regulate toward negativity (blue, see Figure 2) or toward positivity (red, see Figure 2). We used a feather image (Neurocare group Munich, Germany) that moved automatically on the horizontal axis from left to right as feedback. The feather moved vertically according to EEG potential shifts produced by the participant. Participants were instructed to keep the feather above or below the black line. However, we did not provide instructions on how to produce such brain activation. Feather height represented the strength of SCP modulation during the trial.

One trial lasted 13 s, beginning with a 2-s baseline assessment of EEG activity. After 8 s of one trial, a reward screen was presented in case the participant successfully regulated brain activity in the required direction. The reward screen was a bright yellow shining sun. We defined a successful trial as a regulation toward the required polarity for more than half of the trial duration (>4 s).

In sessions one to three, the number of positivity and negativity trials was equal (50% each). Beginning with session four, 60% of the trials were negativity trials, and with the seventh session, 80% were negativity trials. We did not implement more than 80% of negativity trials to rule out any potential adverse effects caused by repeated and intense shifts toward only negativity.

**Figure 3:**
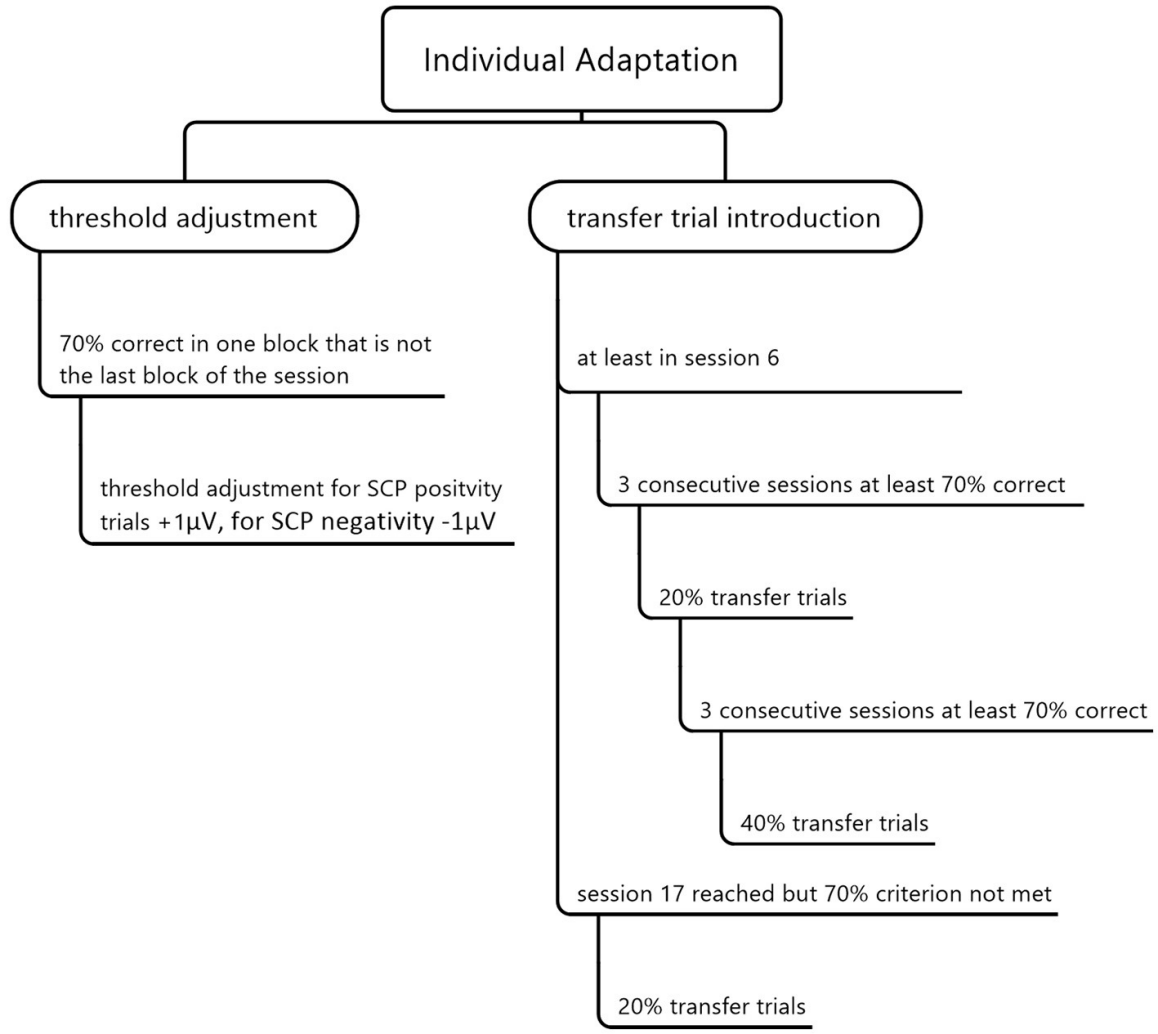
Individual adaptation of the SCP neurofeedback training by threshold adjustment and transfer trial introduction.

We realized individual adaptation by increasing difficulty via threshold adaptation according to participants' performance and introducing transfer trials without online feedback. We started the protocol without setting an individual threshold. Thus, each SCP brain activation shift toward positivity or negativity different from baseline was classified as successful regulation. If a participant successfully regulated toward SCP positivity or negativity in 70% of all trials in one run, the threshold was raised by 1 μV (see Figure 3). We chose 70% as the success criterion as this equals a performance significantly above the chance level, with 14 out of 20 successful trials (*p* = 0.01, Bortz, 1989). Threshold adjustment was separate for positivity (+1 μV) and negativity trials (−1μV), meaning that this 70% correct performance level could be reached or failed independently for positivity and negativity. If a participant performed 70% or above in the last run of one session, we did not increase the threshold in the following session, as each threshold increase should be trained for at least one run during the same session (see Figure 3).

We also included transfer trials in which the desired direction of the EEG activation shift is indicated, but no feedback is provided. Our goal was to strengthen the ability of SCP regulation without being connected to a neurofeedback device. We presented 20% of all trials as transfer trials if a participant performed at least 70% correctly in three consecutive sessions but earliest in session six (see Figure 3). If, after that, 70% of all trials were correct in three consecutive sessions, we increased the transfer trial rate to 40%, which was also the highest possible amount of transfer trials. In session 17, 20% of all trials were introduced as transfer trials for all participants independent of their performance. Participants received training cards for home use after introducing transfer trials to support a behavioral change beyond the effect of transfer trials. The cards showed the screen images of SCP positivity or negativity. The training cards were in check card format and laminated such that it was possible to put them in the purse and use them in daily life waiting situations (e.g., waiting at the doctor's).

Additionally, we instructed participants to practice SCP neurofeedback training in a calm environment without disturbance for at least 20 min a day. We also recommended continuing this home training schedule until the follow-up assessment. All participants agreed to continue SCP regulation training of at least 20 min daily.

EEG was recorded from an Ag/AgCl 12 mm electrode from Cz with a reference on the right mastoid using a TheraPrax amplifier (Neurocare group Munich, Germany) and a sampling rate of 128 Hz. After EEG preparation, we waited 10 min before SCP neurofeedback training started to avoid artifacts by DC drifts. The impedance was below 5 kΩ.

#### 2.3.4. Data analysis

EEG was low pass filtered (10 Hz). Data were segmented based on SCP modulation (positivity/negativity), and each segment was 2 s baseline corrected. Ocular artifacts were corrected using a regression-based approach (Gratton et al., 1983), and the remaining artifacts were excluded with a threshold of ±70 μV. All trials belonging to one condition were averaged. The mean activation of SCP amplitudes was calculated between 1 second and 8 seconds of the feedback trial. Values were exported for statistical analysis using SPSS IBM^®^ version 26. For participant 01, session 10 is missing due to technical problems during data acquisition. For participant 07, we only have data from 14 sessions. In the case of average calculations beyond session 14, thus, the average is based on four participants.

For participant 01, we collected data from the TAP tests for data assessments t0–t3. However, the TAP test Sustained Attention could not be performed at baseline nor t3 due to the exhaustion of the participant. Also, the psychological tests were not assessed at the follow-up assessment as relatives canceled participation before all data were assessed. In participant 03, we could not collect a baseline measurement as there was an urgent wish to immediately start with the training and the decision not to participate otherwise.

As the rate of transfer trials was initially 20%, we only had six transfer trials per run. Therefore, our transfer trial averages were based on 24 trials per session and participant.

As we provide data from *n* = 5 participants, our data analysis is primarily descriptive and focused on individual cases. We considered three criteria for judging the ability of willful SCP regulation: The first was the increase of mean SCP negativity over time. The second one was the fulfillment of the threshold criterion. A participant who reached that criterion must have been successful in 70% of all trials in one run (indicating significance, see Section 2.3.3). The third criterion was the ability to reach transfer trials before session 17, as performance must have been 70% or higher in three consecutive sessions to reach that criterion.

To judge whether performance in the attention domain significantly improved, we used the T value differences predefined in the TAP test manual (Zimmermann and Fimm, 2012). Critical T value differences which indicate significant improvement on a single case level according to Lienert and Raatz (1998) with α = 0.05 were provided in the manual, and we compared those to the T value differences we found in our participants. To judge whether intraindividual changes in the psychological questionnaires were significant, we calculated critical differences (Dcrit) using the Lienert (1961) formula with *SD* = standard deviation of the norm data, *rtt*1 = reliability, and *rtt*2 = re-test reliability.


$$\begin{array}{l} Dcrit\;=\;z*SD*\sqrt{2}-\left(rtt1+rtt2\right)\; \end{array}$$

We used *z* = 1.96, which equals α = 0.0,5, and z-transformed questionnaire data to judge whether the critical difference was exceeded. For correlation calculations, we used Kendall's tau coefficient.

## 3. Results

### 3.1. SCP regulation

**Figure 4:**
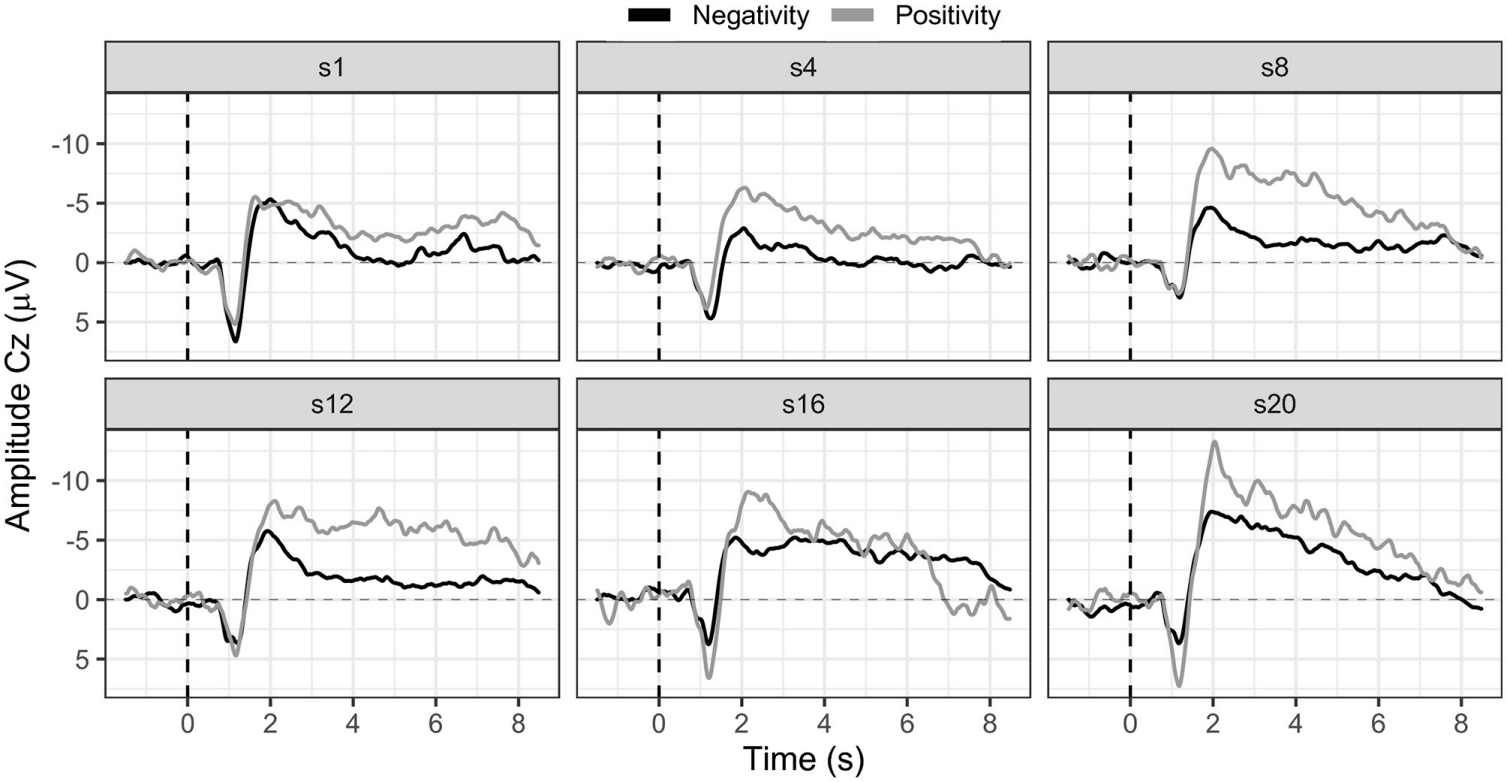
SCP negativity and positivity averaged for all participants for sessions one, four, eight, 12, 16, and 20.

Participants were motivated to learn SCP negativity regulation; however, they mostly performed three runs (120 trials, 18 transfer trials) instead of the planned four runs (160 trials, 24 transfer trials) per session due to increasing exhaustion. On average, participants learned to regulate their SCPs toward negativity. Their mean SCP negativity increased from −1.49 μV to −3.53 μV (see Figure 4). Due to the small sample, we used a non-parametric Friedman ANOVA with SCP negativity as the dependent variable and found a significant change in SCP negativity over sessions [*F*_(19)_ = 32.60, *p* = 0.03], and pairwise comparisons revealed a significant increase between session one and 20 [*F*_(1)_ = 13.50, *p* = 0.02]. Participants were unable to learn SCP positivity regulation. They regulated toward negativity during positivity trials in the first session (session one: −3.26 μV) and increased negativity throughout the training (session 20: −5.85 μV).

Hypothesis one was confirmed as patients on a group level learned to regulate SCP negativity. However, we considered a single case analysis necessary and appropriate.

#### 3.1.1. Strategies for SCP regulation

Overall, strategies such as motor imagery, mental calculation, and mental repetition of lists (months or days) were reported for SCP regulation toward negativity (activation task). For regulation toward positivity (deactivation task), strategies such as inner calmness and relaxation were reported. However, some participants could not describe specific strategies. Again we believed a single case report to be valuable and added individual strategies to the SCP regulation case reports.

#### 3.1.2. Participant 01

**Figure 5:**
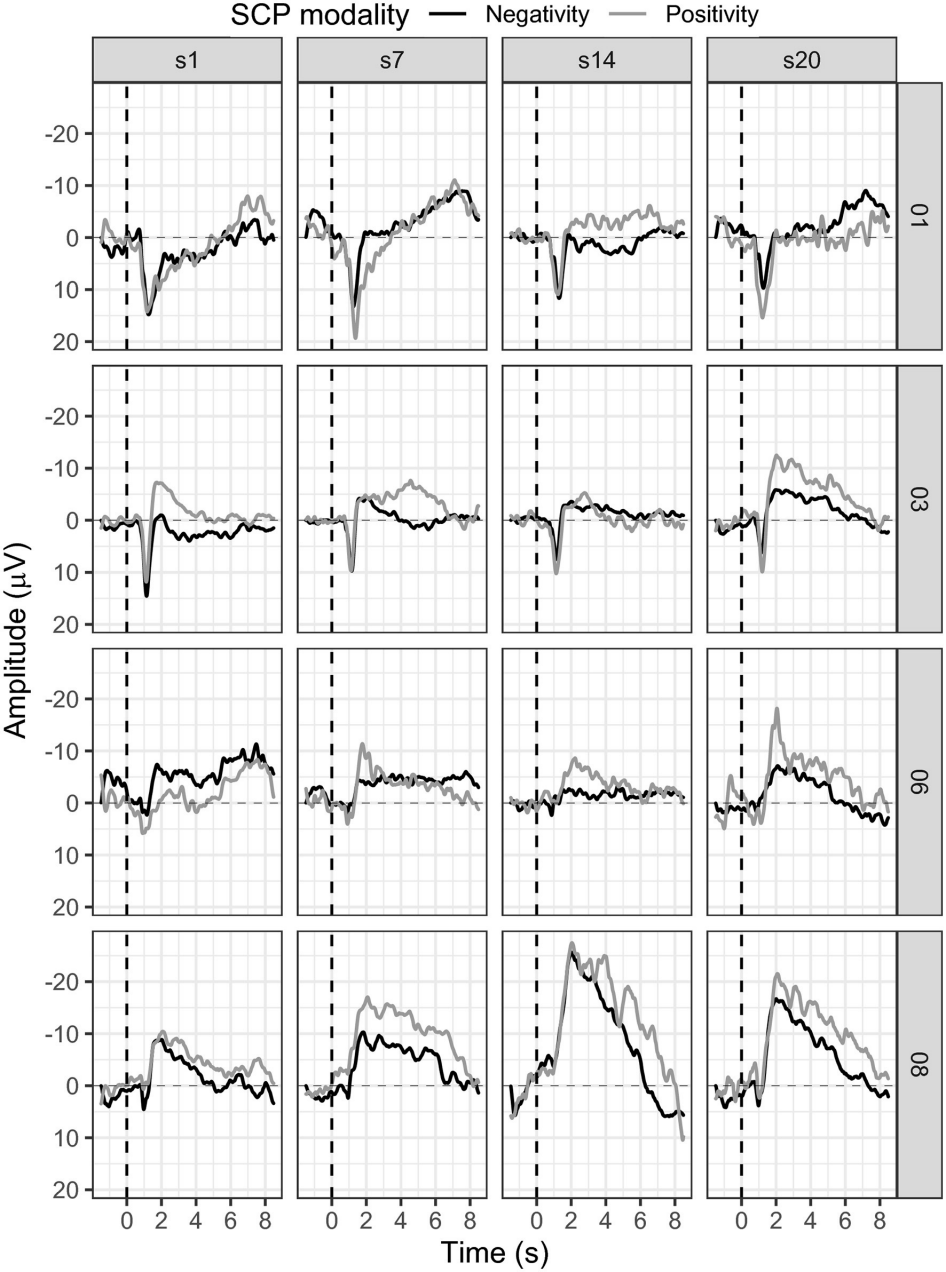
Mean SCP activity for participants 01, 03, 06, and 08 in sessions one, seven, 14, and 20.

In session one, the mean SCP negativity was −1.47 μV and improved to −3.35 μV in session 20 (see Figure 5). Participant 01 reached a training threshold increase for negativity once in session 13. SCP positivity mean activity was 0.43 μV in session 1 and −0.35 μV in session 20 (see Figure 5). The criterion to increase the training threshold was not met during positivity trials.

In session three, the participant reported having forgotten the meaning of the triangles on the feedback screen. Therefore, she used the strategy for positivity (triangle downwards) and during negativity trials (triangle upwards) which led to regulation toward positivity during this session. After session nine, the participant also reported a change in the strategy for SCP negativity. Until session nine, she had mentally recited the days of a week or the months of a year. Beginning in session nine, she started alternating both strategies with every trial. For positivity, no active strategy could be explained. However, this change in strategy did not lead to stable SCP negativity control as the initial success in sessions 11 and 12 was followed by mean activation values in the positivity range in sessions 13, 14, and 15.

**Figure 6:**
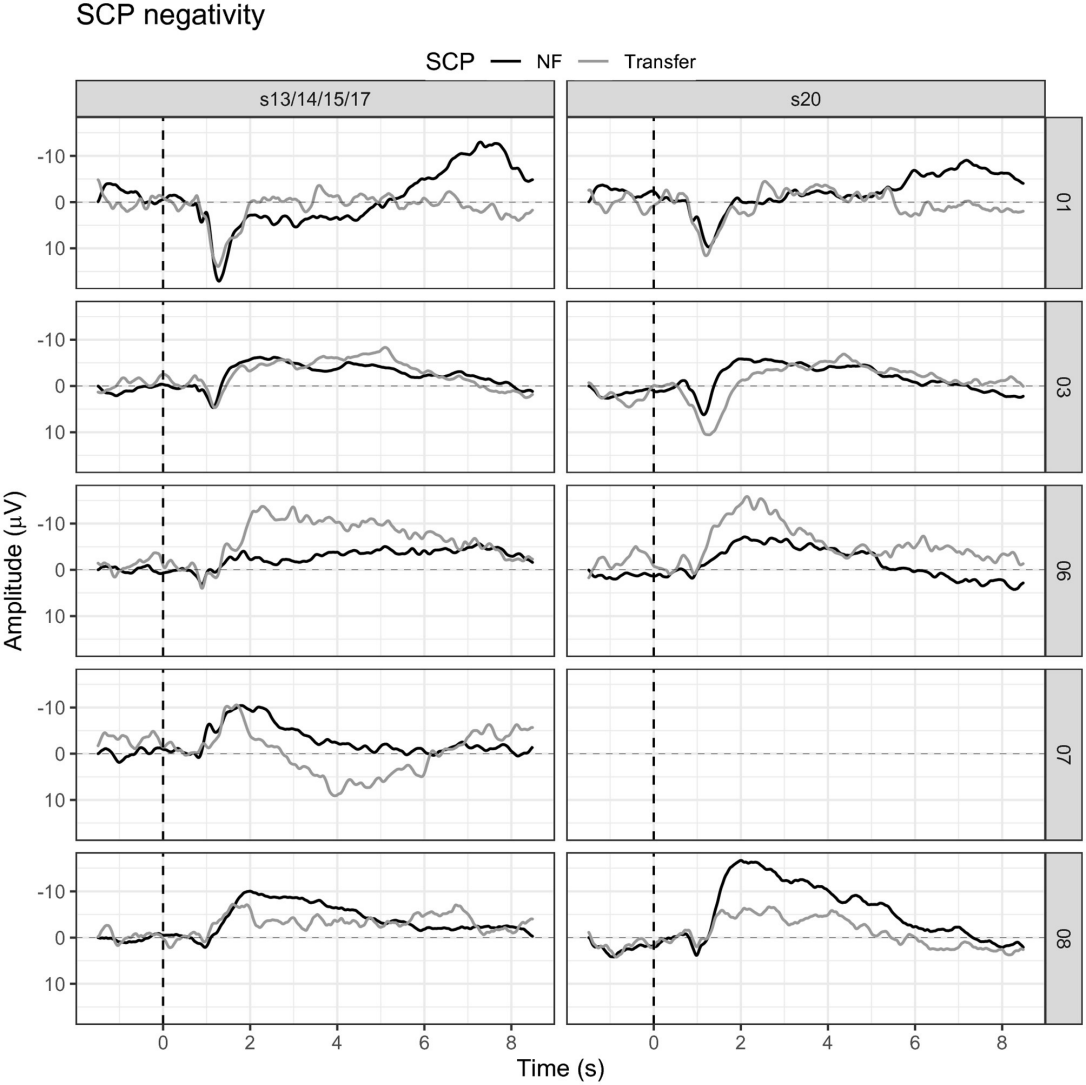
Mean SCP negativity during feedback and transfer trials for all participants in the first session in which transfer trials were introduced, and in the last training session.

Transfer trials were introduced in session 15 and participant 01 perceived those as more manageable and less disturbing than regular feedback trials (see Figure 6). However, mean SCP negativity activation was more successful during feedback trials (session 15 = −1.84 μV, session 20 = −3.35 μV) as compared to transfer trials (session 15 = 0.48 μV, session 20 = −0.07 μV).

Participant 01 successfully increased SCP negativity throughout the training, reaching one threshold adjustment and introducing transfer trials before session 17. Taken together, these results indicate successful SCP control toward negativity.

#### 3.1.3. Participant 03

Participant 03 continuously enhanced SCP negativity regulation (see Figure 5). With some outliers in sessions 10, 11, 12, and 18, SCP negativity regulation improved over time. For example, while the mean SCP negativity in session one was 1.21 μV, it improved to −3.26 μV in session 20 (see Figure 5). In addition, in session one, the negativity training threshold was increased the first time; and in session two, it was increased a second time and stayed the same.

Regulation of SCP positivity was unsuccessful as mean activity increased in negativity over sessions. While in session one, the mean activity for SCP positivity was −1.68 μV, it was −5.92 μV in session 20 (see Figure 5). No threshold adjustment was reached.

The participant could not report strategies for the SCP activation task (negativity) and reported following an inner feeling on producing the required brain activation. Beginning in the fourth session, he reported problems in the deactivation task (positivity task) and felt under pressure to force positivity activation. Therefore, actively trying not to follow the movement of the feedback feather was the new strategy. This strategy was successful in sessions five and nine and led to more stable SCP control after session 12.

Participant 03 was the only participant in the study who started with transfer trials in session 13 already (see Figure 6). Mean SCP negativity during feedback trials in session 13 was −3.15 μV, while in session 20, it was −3.26 μV. In the transfer trials, SCP negativity was even more prominent, with −3.39 μV in session 13 and a slight decrease to −2.34 μV in session 20.

Participant 03 successfully increased SCP negativity over time, reached two threshold adjustments, and introduction of transfer trials before session 17. Therefore, we conclude that participant 03 learned to regulate SCP negativity.

#### 3.1.4. Participant 06

In participant 06, average SCP negativity was highest at the beginning of the training during sessions one and three (see Figure 5). While in session one, mean SCP negativity activation was −6.56 μV, it was on average −1.87 μV in session 20. Two threshold adjustments were reached in SCP negativity, one in session nine and one in session 11. In session four, the participant successfully regulated SCP negativity until session 11. After the second threshold adjustment, SCP negativity regulation was more challenging between sessions 14 to 20. SCP positivity increased toward negativity over time with a mean activation of −2.86 μV in session one and −5.69 μV in session 20 (see Figure 5). SCP positivity regulation was not successful.

In sessions one to four, the participant followed an inner strategy to influence the feather without being able to describe the strategy in detail. Beginning in session five, the participant tried mental calculation for the activation task (negativity) and relaxation for the deactivation task (positivity). These strategies were subjectively not being experienced as successful, leading to the participant's growing frustration. After session 14, the participant requested an extra appointment as the feeling of losing control was too overwhelming. The therapist tried not to dictate strategies but to encourage the participant to follow their thoughts when trying to find a new strategy. The participant introduced the idea of trying to revive old memories of events rarely thought about as a strategy for activation, which was used for sessions 16 to 20. In sessions 17 to 20, the participant reported a higher feeling of control for negativity trials but less for positivity trials. Overall, this participant was exhausted by the training and suffered from the intense effort by showing signs of fatigue and tiredness (longer breaks required, yawning, and feeling sleepy), and frustration.

Transfer trials were started in session 17. Participant 06 had reached the criteria to start transfer trials in the same session in which the protocol would have introduced transfer trials anyway. While mean SCP negativity during feedback trials was −3.42 μV in session 17, it was −8.08 μV in session 17 for transfer trials. In session 20, the mean SCP negativity during feedback trials was −1.87 μV, while it was−6.28 μV in transfer trials (see Figure 6).

Participant 06 did not continuously increase SCP negativity throughout the neurofeedback therapy. Transfer trials were particularly successful but were introduced late in the training. We cautiously conclude that participant 06 learned SCP modulation.

#### 3.1.5. Participant 07

**Figure 7:**
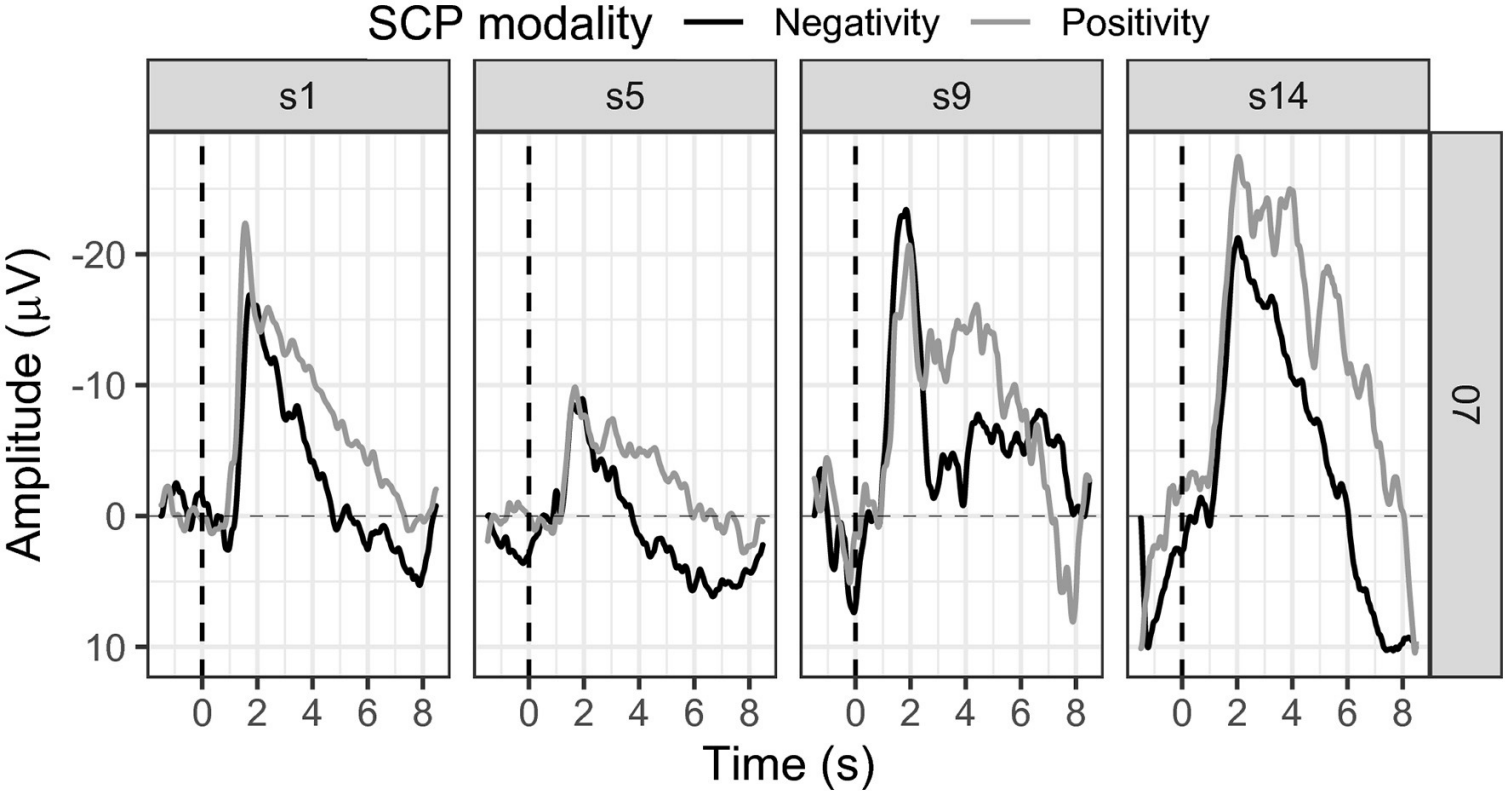
Mean SCP activity for participant 07 in sessions one, five, nine, and 14.

SCP negativity regulation slightly improved throughout the sessions (see Figure 7). While in session one the mean SCP negativity was −2.80 μV, it was −2.84 μV in session 14 (see Figure 7).

The first threshold adjustment was reached in session five, another in session 12, and the last in session 13. SCP positivity showed a mean activity in the first session of −7.32 μV, stayed in the negativity range throughout the training, and increased to −14.18 μV in session 14 (see Figure 7). Positivity could not be successfully regulated, and no threshold adjustment was reached.

Participant 07 reported mental calculation as a strategy for the activation task. Concerning SCP positivity, participant 07 could not report any conscious strategy. Transfer trials were introduced in session 14 as we already knew then that the participant could not finish the training with 20 sessions. Mean SCP negativity was −2.84 μV for feedback trials and 0.41 μV in transfer trials (see Figure 6).

We cautiously conclude successful SCP negativity regulation, as this participant achieved three threshold adjustments in only 14 sessions. However, the number of sessions was reduced, and transfer trials were introduced in the final session independent of the preset performance criteria.

#### 3.1.6. Participant 08

Participant 08 learned to regulate SCP negativity over time. While in session one, the average activity was −2.81 μV, it improved to a mean activity of −6.64 μV (see Figure 5). The first threshold adjustment was reached in session five, the second in session seven, the third in session 11, and the last threshold increase to −4 μV was adjusted in session 14. However, SCP positivity was not learned, and mean activity was −4.86 μV in session one and −11.44 μV in session 20 (see Figure 5).

Participant 08 reported the imagination of chaotic movements by humans and animals for the activation task (negativity) and a feeling of inner calmness for the deactivation task (positivity). The participant reported intuitively developing these strategies without active effort to create a strategy. The inner imagination of chaotic movements remained the strategy during the neurofeedback sessions.

Transfer trials were introduced in session 15. In session 20, the percentage of transfer trials could be increased to 40%. Average SCP negativity in session 15 was −4.75 μV in feedback trials and −3.67 μV in transfer trials (see Figure 6). In session 20, a mean SCP negativity of −6.64 μV was achieved; and in the transfer trial, the mean activation was −1.34 (see Figure 6).

Taken together, participant 08 was successful in SCP regulation toward negativity. Mean SCP negativity increased and four threshold adjustments as well as a percentage of 40% concerning transfer trials were achieved.

### 3.2. Attention

All participants reported practicing the newly acquired SCP regulation skill at home between t2 and t3, supported by the learning cards. Participant 07 especially emphasized the amount of time spent practicing. Participant 08 acknowledged not having practiced intensely between t2 and t3 due to many leisure time activities and a more extended holiday in this period.

#### 3.2.1. Participant 01

Participant 01 significantly improved alertness reaction times with and without signal between the post-SCP training assessment and the follow-up measurement (see Table 2). Concerning divided attention, we found an improvement between t1 and t2, which stayed stable between t2 and t3. Furthermore, the errors decreased significantly between t1 and t2 and even more between t2 and t3 (Table 2). For the Sustained Attention TAP test, we found significantly faster reaction times after as compared to before SCP training; however, this test could not be performed t0 nor t3 due to the exhaustion level of the participant on the measurement day. Reaction time in the Go/NoGo task improved significantly between t2 and t3.

**Table 2:** Behavioral measures for the assessment of attention in participant 01.

**TAP**	**T0**		**T1**		**T2**		**T3**		**T diff**			**CTD**
	**M**	**T**	**M**	**T**	**M**	**T**	**M**	**T**	**T0-T1**	**T1-T2**	**T2-T3**	
**Alert**												
Wo sig	863	< 20	977	< 20	818	< 20	274	42	0	0	**22**	3.72
Sig	910	< 20	729	< 20	628	< 20	264	41	0	0	**21**	4.50
	**No**	**T**	**No**	**T**	**No**	**T**	**No**	**T**				
**DA**												
Omiss	3	42	2	46	0	61	0	61	4	**15**	0	13.06
Err	6	34	10	31	3	39	0	61	−3	**8**	**21**	7.59
	**M/No**	**T**	**M/No**	**T**	**M/No**	**T**	**M/No**	**T**				
**SA**												
RT	-	-	930	25	754	38	-	-	-	**13**	-	6.36
Omiss	-	-	11	49	14	46	-	-	-	−3	-	10.31
Err	-	-	11	41	6	48	-	-	-	7	-	10.76
**GNG**												
RT	990	< 20	932	< 20	661	26	451	47	0	6	**21**	8.03
Omiss	2	33	3	29	1	38	0	>39	−4	9	1	14.22
Err	0	>53	0	>53	0	>53	0	>53	0	0	0	15.75

TAP, test battery for attention performance; t0, baseline; t1, pre training; t2, post training; t3, follow-up; Tdiff, T value difference; CTD, critical T value difference; M, mean; T, T norm value; No, number; Alert, Alertness; wo sig, without signal; sig, with signal; DA, divided attention; omiss, omissions; err, errors; SA, sustained attention; RT, reaction time; GNG, GoNoGo; -, missing data. Bold numbers indicate significant changes.

Attention performance improved in participant 01. Alertness, divided attention, and the Go/NoGo performance improved between t2 and the follow-up assessment.

#### 3.2.2. Participant 03

Participant 03 improved divided attention significantly in omissions between t1 and t2. However, this improvement was not stable, and at follow-up, the original performance level was reached (see Table 3). In the Go/NoGo task, reaction time was improved between t1 and t2 and stayed stable at t3. However, this patient's baseline assessment was missing; therefore, comparing t0 and t1 was impossible.

**Table 3:** Behavioral measures for the assessment of attention in participant 03.

**TAP**	**T0**		**T1**		**T2**		**T3**		**T diff**			**CTD**
	**M**	**T**	**M**	**T**	**M**	**T**	**M**	**T**	**T0-T1**	**T1-T2**	**T2-T3**	
**Alert**												
Wo sig	-	-	247	48	232	50	247	48	-	2	−2	3.72
Sig	-	-	248	46	234	47	243	46	-	1	−1	4.50
	**No**	**T**	**No**	**T**	**No**	**T**	**No**	**T**				
**DA**												
Omiss	-	-	2	46	0	63	2	46	-	**17**	**-17**	13.06
Err	-	-	11	31	5	35	9	32	-	4	−3	7.59
	**M/No**	**T**	**M/No**	**T**	**M/No**	**T**	**M/No**	**T**				
**SA**												
RT	-	-	574	52	539	55	541	55	-	3	0	6.36
Omiss	-	-	7	63	6	67	6	68	-	4	1	10.31
Err	-	-	4	51	3	54	9	44	-	3	−10	10.76
**GNG**												
RT	-	-	421	56	380	64	550	62	-	**8**	−2	8.03
Omiss	-	-	0	>39	1	38	0	>34	-	1	−4	14.22
Err	-	-	1	52	1	52	1	47	-	0	−5	15.75

TAP, test battery for attention performance; t0, baseline; t1, pre training; t2, post training; t3, follow-up; Tdiff, T value difference; CTD, critical T value difference; M, mean; T, T norm value; No, number; Alert, Alertness; wo sig, without signal; sig, with signal; DA, divided attention; omiss, omissions; err, errors; SA, sustained attention; RT, reaction time; GNG, GoNoGo; -, missing data. Bold numbers indicate significant changes.

In participant 03, no stable improvement in attention could be found throughout this study.

#### 3.2.3. Participant 06

Participant 6 improved significantly in the TAP test's alertness and divided attention between t0 and t1. Concerning alertness with a warning signal, another significant improvement was found between t1 and t2, but advances declined to the level before SCP training between t2 and the follow-up assessment (see Table 4). Finally, the error rate increased between t1 and t2 in the sustained attention task and remained stable at t3.

**Table 4:** Behavioral measures for the assessment of attention in participant 06.

**TAP**	**T0**		**T1**		**T2**		**T3**		**T diff**			**CTD**
	**M**	**T**	**M**	**T**	**M**	**T**	**M**	**T**	**T0-T1**	**T1-T2**	**T2-T3**	
**Alert**												
Wo sig	314	36	262	46	267	45	265	44	**10**	−1	−1	3.72
Sig	316	35	277	42	243	47	271	42	**7**	**5**	−5	4.50
	**No**	**T**	**No**	**T**	**No**	**T**	**No**	**T**				
**DA**												
Omiss	3	44	0	63	0	63	3	44	**19**	0	−19	13.06
Err	9	32	5	35	10	31	15	30	3	−4	–**1**	7.59
	**M/No**	**T**	**M/No**	**T**	**M/No**	**T**	**M/No**	**T**				
**SA**												
RT	659	49	574	50	650	48	623	48	1	−2	0	6.36
Omiss	19	43	22	41	20	42	14	48	−2	1	6	10.31
Err	19	34	20	32	31	< 20	30	< 20	−2	–**12**	0	10.76
**GNG**												
RT	463	45	468	46	452	52	447	52	1	6	0	8.03
Omiss	0	>39	0	>39	0	>39	1	33	0	0	−6	14.22
Err	0	>53	0	>53	1	52	1	47	0	1	0	15.75

TAP, test battery for attention performance; t0, baseline; t1, pre training; t2, post training; t3, follow-up; Tdiff, T value difference; CTD, critical T value difference; M, mean; T, T norm value; No, number; Alert, Alertness; wo sig, without signal; sig, with signal; DA, divided attention; omiss, omissions; err, errors; SA, sustained attention; RT, reaction time; GNG, GoNoGo; -, missing data. Bold numbers indicate significant changes.

In participant 06, we conclude no improvement in attention performance during this study.

#### 3.2.4. Participant 07

Participant 07 improved divided attention between t2 and t3 in both parameters, omissions and errors. Similarly, omission rates in sustained attention improved between t2 and t3 (see Table 5). Alertness performance decreased significantly between t1 and t2 and remained lower until t3 (see Table 5). The Go/NoGo performance did not change with the SCP training. We conclude that a positive effect on divided and sustained attention occurred mainly after the SCP training until the follow-up assessment.

**Table 5:** Behavioral measures for the assessment of attention in participant 07.

**TAP**	**T0**		**T1**		**T2**		**T3**		**T diff**			**CTD**
	**M**	**T**	**M**	**T**	**M**	**T**	**M**	**T**	**T0-T1**	**T1-T2**	**T2-T3**	
**Alert**												
Wo sig	240	55	243	55	267	45	276	48	0	**−10**	3	3.72
Sig	221	57	220	58	243	47	243	50	1	**−11**	3	4.50
	**No**	**T**	**No**	**T**	**No**	**T**	**No**	**T**				
**DA**												
Omiss	1	52	1	52	3	43	0	63	0	−10	**20**	13.06
Err	6	34	7	33	10	31	2	44	−1	−2	**13**	7.59
	**M/No**	**T**	**M/No**	**T**	**M/No**	**T**	**M/No**	**T**				
**SA**												
RT	664	45	644	47	616	49	581	50	2	2	1	6.36
Omiss	30	31	13	49	13	49	6	68	**18**	0	**19**	10.31
Err	4	51	7	47	7	47	6	48	−4	0	1	10.76
**GNG**												
RT	494	41	455	47	485	45	557	41	6	−2	−4	8.03
Omiss	0	>39	0	>39	0	>39	0	>39	0	0	0	14.22
Err	3	41	1	52	1	47	1	52	11	−5	5	15.75

TAP, test battery for attention performance; t0, baseline; t1, pre training; t2, post training; t3, follow-up; Tdiff, T value difference; CTD, critical T value difference; M, mean; T, T norm value; No, number; Alert, Alertness; wo sig, without signal; sig, with signal; DA, divided attention; omiss, omissions; err, errors; SA, sustained attention; RT, reaction time; GNG, GoNoGo; -, missing data. Bold numbers indicate significant changes.

#### 3.2.5. Participant 08

After the SCP training, participant eight reacted significantly faster in the alertness task with and without a signal. This improvement disappeared until the follow-up measurement, and the participant showed almost the t0 performance. In the divided attention, a similar effect occurred with significantly fewer omissions between t0 and t1 but a final performance level equaling t0 performance. Interestingly the ability to avoid errors improved significantly between t2 and t3. Concerning sustained attention, reaction times and omissions improved between t1 and t2, but both effects vanished until the t3 assessment. The participant made significantly fewer errors at t1 as compared to t0 but then again significantly more errors at t2 than at t1. At the t3 assessment, the ability to avoid errors was, again, significantly improved. Reaction times in the Go/NoGo task improved between t1 and t2, but the effect remained stable until t3 (see Table 6).

**Table 6:** Behavioral measures for the assessment of attention in participant 08.

**TAP**	**T0**		**T1**		**T2**		**T3**		**T diff**			**CTD**
	**M**	**T**	**M**	**T**	**M**	**T**	**M**	**T**	**T0-T1**	**T1-T2**	**T2-T3**	
**Alert**												
Wo sig	486	28	518	26	258	41	480	29	−2	**15**	**−12**	3.72
Sig	520	24	273	27	250	44	535	24	3	**17**	**−20**	4.50
	**No**	**T**	**No**	**T**	**No**	**T**	**No**	**T**				
**DA**												
omiss	3	44	0	63	1	51	3	44	**19**	−12	−7	13.06
Err	9	32	5	35	8	32	1	52	3	−3	**20**	7.59
	**M/No**	**T**	**M/No**	**T**	**M/No**	**T**	**M/No**	**T**				
**SA**												
RT	953	24	928	29	757	41	921	32	5	**12**	–**9**	6.36
Omiss	31	< 20	36	< 20	26	31	25	< 20	0	**11**	–**11**	10.31
Err	30	< 20	5	35	63	< 20	4	37	**15**	–**15**	**17**	10.76
**GNG**												
RT	485	39	519	37	373	56	564	33	−2	**19**	–**23**	8.03
Omiss	0	>39	0	>39	0	>39	0	>39	0	0	0	14.22
Err	1	52	0	>53	1	47	0	>48	1	−6	1	15.75

TAP, test battery for attention performance; t0, baseline; t1, pre training; t2, post training; t3, follow-up; Tdiff, T value difference; CTD, critical T value difference; M, mean; T, T norm value; No, number; Alert, Alertness; wo sig, without signal; sig, with signal; DA, divided attention; omiss, omissions; err, errors; SA, sustained attention; RT, reaction time; GNG, GoNoGo; -, missing data. Bold numbers indicate significant changes.

Considering the results in all patients, decision speed (Go/NoGo reaction time), sustained attention, and divided attention parameters mostly improved after as compared to before SCP training. However, these improvements were only stable in some participants in the follow-up measurement. Therefore, H2 of SCP NF improving attention performance was only partially confirmed.

### 3.3. Emotional state and quality of life

To judge emotional state and quality of life, we compared the results of our psychological tests for t0, t1, t2, and the follow-up assessment t3.

#### 3.3.1. Depression—ADS

**Figure 8:**
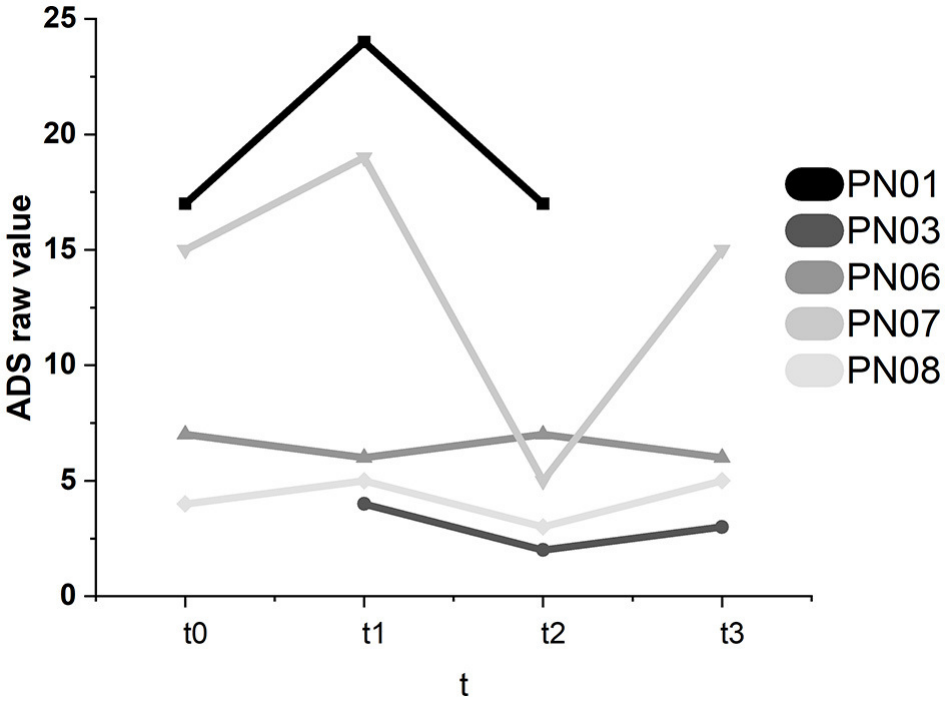
ADS raw values in all participants for all assessments.

Participant 07 showed significant intraindividual changes in the ADS scores (see Figure 8). The ADS score dropped significantly between t1 and t2 (−14). At t3, the value significantly increased to the t0 level (+10). Therefore, in participant 07, there might have been a short-term positive effect of SCP NF, which did not lead to a stable improvement in emotional state. In participant 01, the ADS raw value increased between baseline and t1 (+7). It decreased again to the original value at t2 (−7). Therefore, the decrease between t1 and t2 lies in the individual range of variation and cannot be attributed to the SCP NF intervention. These changes were not significant.

#### 3.3.2. Self-efficacy—ASS

Global self-efficacy was constant and was not majorly influenced by the SCP NF intervention. Except for participant 01, whose global self-efficacy ranged between percentile ranks 20 and 40, subjective self-efficacy was judged high in the other participants, with values ranging between percentile rank 70 and above 98 for all assessments.

Interestingly, in participant 06, there was an increase in work- and performance-related self-efficacy from percentile rank 50 at t0 and t1 to percentile rank 60 at t2 and 90 at t3. In participants 03, 07, and 08, work- and performance-related self-efficacy ranged between 50 and above 98 and therefore was relatively high. In participant 01, the percentile rank was initially 30 and stayed the same.

**Figure 9:**
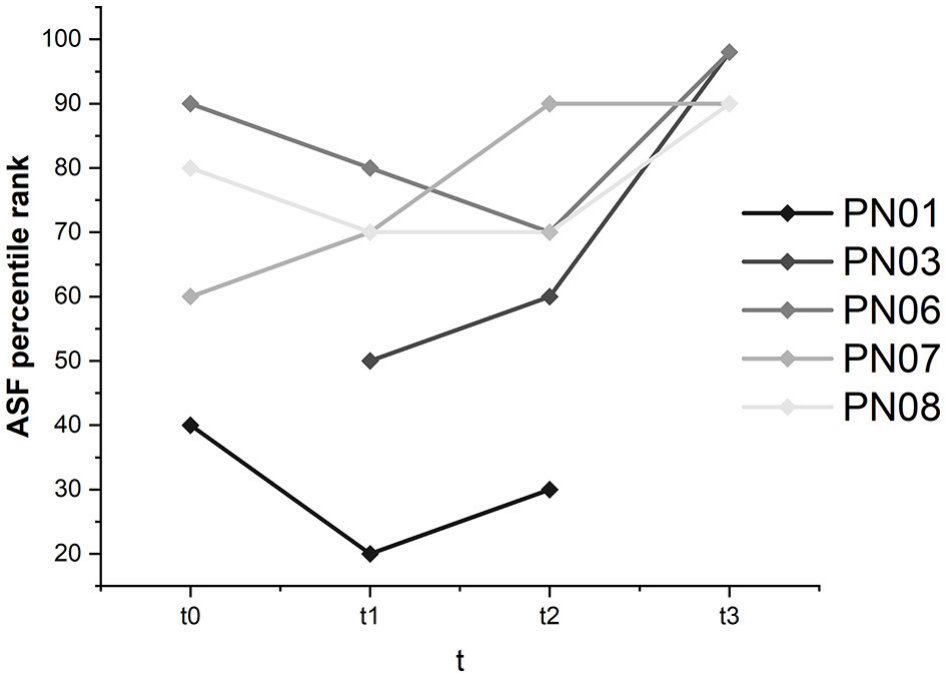
Percentile ranks for all participants in the ASF interaction domain.

Participants 03, 06, 07, and 08 judged health-related self-efficacy as high and stable (ranging between 50 and above 98). Participant 01 considered health-related self-efficacy with a percentile rank of 30 at t0 and t1 and 50 at t2. According to the critical difference value, the only significant change was found for participant 03 in the ASF interaction domain (see Figure 9). Participant 03 judged self-efficacy in the interaction domain with a percentile rank of 50 before the training and reached above 98 at t3.

#### 3.3.3. Activities of daily living—AFIB

**Figure 10:**
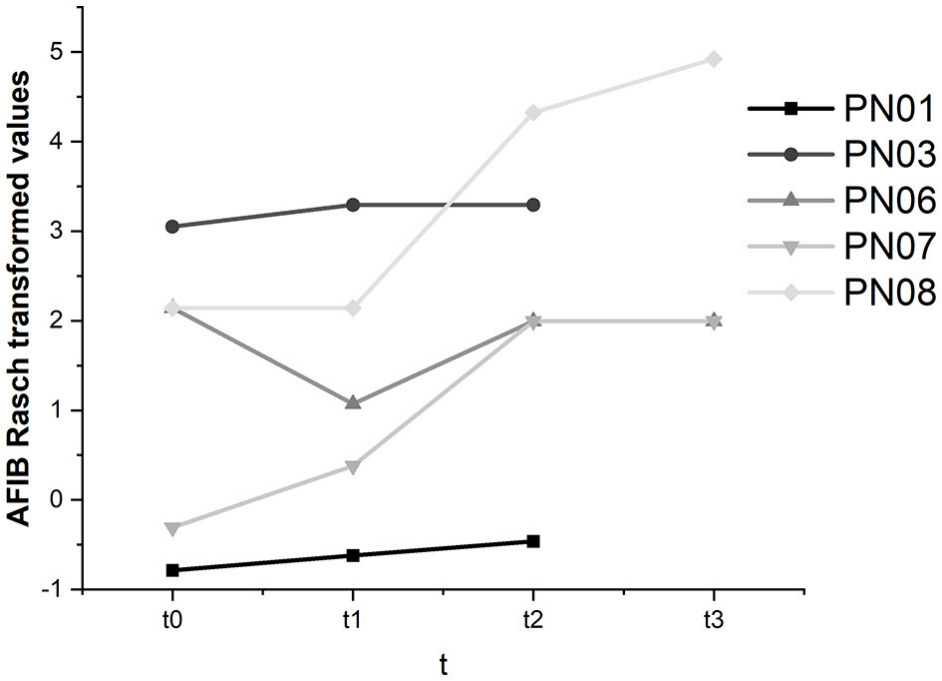
AFIB scores for all participants and all assessments.

As the AFIB questionnaire is not a publicly available instrument and we could not access the required information to calculate critical differences to judge whether intraindividual changes were significant, results were described descriptively (see Figure 10).

In participants 01, 03, 07, and 08, we found an increase in the ability to manage activities of daily living throughout this study. In participant 06, the decline in subjectively perceived participation between baseline and t1 was moderated between t1 and t2 but did not reach the t0 level at t3. Across all participants, AFIB scores improved from 0.35 at t0 to 3.05 at t3.

#### 3.3.4. Quality of life—SF36

We found no significant changes in the physical functioning sum score, with an average value of 33.83 at t0 and a value of 39.20 at t3. Even though the t3 value was close to the normal range (40–60) and improved compared to the baseline, it remained below the average range threshold. The mental health score was higher on average at t0 (52.17) and t3 (46.99) and within the normal range for all participants for all assessments. In participant 07, there was a significant drop in the mental health sum score between baseline (58.47) and t1 (41.51). However, values significantly improved at t2 (52.56) and remained stable at t3 (53.10). The fact that the significant changes ranged in the individual variation of the patient showed that changes were not attributable to the SCP NF intervention.

Taken together, we found an increase in the performance of activities of daily living and increased self-efficacy in the interaction domain. However, results could be more consistent, so H3 of psychological variables improved by SCP NF was only partially confirmed.

### 3.4. Motivation and attention and their influence on SCP negativity regulation

In participant 03, we found a significant correlation (Kendall's tau) between motivation and SCP negativity. The more motivated the participant was, the more negative the SCP amplitude (*r* = −0.38, *p* = 0.04). In participant 08, we also found the same significant relation between motivation and SCP negativity (*r* = −0.45, *p* = 0.04). Subjectively reported attention was not significantly correlated to SCP negativity.

As only two out of five participants' motivation and SCP negativity were related, our fourth hypothesis (H4) was only partially confirmed.

## 4. Discussion

This study investigated whether patients with chronic attention deficits after stroke can learn to increase attention performance via SCP NF therapy. We found that stroke is a manageable obstacle to successful SCP regulation. All participants learned SCP control toward negativity. However, none of our participants learned SCP regulation toward positivity. Of course, with only 20% of all trials being SCP positivity trials (6 trials per run), learning was complex, and it seemed as if participants were regulating toward negativity in all trials. In this study, our goal was not to equally train positivity and negativity as we focused on the possible behavior change accompanying SCP negativity (Elbert, 1993). However, future studies might include quantitative EEG (Arns et al., 2012) to individually adapt the NF therapy such that negativity and positivity might be more balanced and possible post-stroke changes in cortical excitability are considered (Clarkson and Carmichael, 2009).

We found a high interindividual variation concerning the strategies implemented to gain SCP control. While some participants reported mental calculation, others tried to remember events of personal importance. Mental calculation is one of the more reliable strategies for brain activity control (Friedrich et al., 2013). Autenrieth et al. (2020) reported in their study on NF strategies that the “cognition” strategy is most used in responders and is one of the more effective strategies. This strategy includes all thoughts and mental processes independent of the NF task. Thus, mental calculation, reciting the months of a year, and personal memories can all be subsumed under this strategy. We also had a participant who could not report any strategy observed before, which might be a sign of automatization of the process (Kober et al., 2013; Davelaar, 2018). Strategies were highly individual, and we cannot distinguish more effective from less effective strategies due to our small sample size.

Our sample size calculation might have been overly enthusiastic, as we assumed a medium treatment effect. To detect the effects of NF training with a small treatment effect, at least 138 participants would be needed, according to Faul et al. (2007). Using the Cohen (1988) method and an effect size of 0.20 within the G^*^Power tool, the calculated sample size would already be 274. Our objective in this study was to investigate the potential benefits of NF neurofeedback therapy for stroke patients, which had not been explored previously. Therefore, commencing with a clinical trial may not have been the most appropriate approach for this research. Unfortunately, we were unable to reach our intended sample size to fully comprehend the topic at hand. The COVID-19 pandemic created obstacles in obtaining a larger group of individuals for assessment, and we were unable to recruit more participants before the project funding was exhausted.

Our participants improved in tests for attention performance. However, improvements were very individual and only sometimes enduring. We found an improvement in reaction times of the Go/NoGo task, which correlates with decision-making speed (Zimmermann and Fimm, 2012). Also, sustained attention improved in some participants; however, there was no typical pattern concerning the test variable to be enhanced (errors, omissions, reaction times). The ability to react faster and more flexibly was trained; however, in this case, more homogenous improvements in the alertness test should have occurred, which we could not find. Interestingly, participants 01 and 07, in whom we found the most gains in attention parameters, also showed improvements in psychological tests. Both participants showed increased self-efficacy in the interaction domain, participant 01 also in the health domain, and both participants reported more participation in activities of daily living. Thus, with a bigger sample, we might have shown improved function due to NF therapy and related improvements in psychological state. Furthermore, it might well be that possible improvements in attention were not fully captured in the TAP tests as from the beginning we combined the SCP training with daily tasks such as trying to increase brain excitability while shopping or before listening to the specialist during a doctor's appointment. Participants reported an improved ability to manage daily activities for which attention allocation was necessary (Sohlberg and Raskin, 1996). Future studies might want to include tests that assess attention network functioning to judge possible improvements differently (Posner and Dehaene, 1994).

In two participants (01 and 07), we found improvements in attention parameters between the post-intervention assessment (t2) and the follow-up assessment (t3) but not between the pre-intervention assessment (t1) and t2. We do believe that such improvement might have occurred due to the additional SCP modulation practice at home with the training cards during these 3 months. One might argue that these improvements occurred because of another event independent of our intervention and, of course, we cannot prove that this is not the case. However, both participants experienced chronic attention deficits for 1 and 7 years, respectively. Both participants reported not having received another treatment during the follow-up period nor having experienced major changes in their life situation. If a TAP training effect was responsible for the improvements, it should have occurred already before t3. Additionally, the shortest time between assessments was 4 weeks (t0 and t1) and the longest was 4 months (t1–t2; t2–t3). Bühner et al. (2006) reported a maximum performance gain due to training effects of 7% for the subtests divided attention and Go/NoGo (Zimmermann and Fimm, 2012). Interestingly, participant 08 who presented decreased values between t2 and t3 in all, but one attention parameter was the only participant who reported not having practiced SCP regulation regularly. On the other hand, this participant was most successful in SCP negativity regulation and one could have expected him to be more successful in attention performance improvement.

We found no change in depressive symptoms or quality of life. However, the SF-36 questionnaire (Morfeld et al., 2012) is very focused on physical functioning and the items summarized for the mental health sum score are very general. We found an improvement in self-efficacy in all participants and a significant change in the domain of interaction in one participant. We cannot judge whether this effect was directly attributable to the SCP NF intervention or to the fact that participants needed to interact with the experimenter in our study. They reported having gained new social competencies by interacting with the experimenter in a research context. As self-efficacy is an important predictor of health status and quality of life after stroke (Jones and Riazi, 2011), it should be supported during rehabilitation and possibly utilizing neurofeedback interventions. Our participants were proud to reach individual thresholds that showed their ability to control brain activity. However, our procedure of increasing individual thresholds also increased the difficulty of the task and might have been too demanding as mostly only three runs per session were performed instead of four due to exhaustion by participants. We still consider individual adaptation important; however, it was very difficult to find participants who were willing to attend so many sessions, not only because of private obligations and appointments but also because of illness or short-term appointments. We believe that a study with a larger sample should involve a medical center for participant recruitment. Then again, we also might have included more participants without the COVID-19 pandemic.

We found self-reported motivation to correlate with SCP negativity in two participants. The more they were motivated, the more negative their mean SCP activity was. We found earlier that brain activity regulation was related to motivation (Kleih-Dahms et al., 2021). But in a sample of stroke patients, the adverse effect was shown that higher motivation led to less brain activation and therefore less performance (Kleih et al., 2013).

## 5. Conclusion

In this study, we successfully showed SCP NF therapy to be feasible in post-stroke patients who report chronic attention deficits. We found indications for improvements in cognitive function, participation in activities of daily living, and self-efficacy. However, our sample size is very small, and interindividual variance is high. Therefore, further studies with larger samples are required, even though recruitment may be challenging with a study protocol that includes 20 sessions or more. SCP NF therapy might be a potential new rehabilitation method for the treatment of chronic post-stroke cognitive deficits and should be subject to future research.

## Data availability statement

The raw data supporting the conclusions of this article will be shared upon reasonable request to the corresponding author.

## Ethics statement

The studies involving human participants were reviewed and approved by Medical Ethics Committee of the University of Würzburg. The patients/participants provided their written informed consent to participate in this study.

## Author contributions

SK-D: design, procedure, data acquisition, data analysis, interpretation, and writing manuscript. LB: data analysis and writing manuscript. All authors contributed to the article and approved the submitted version.
